# Characterization of a Macro- and Micro-Textured Titanium Grade 5 Alloy Surface Obtained by Etching Only without Sandblasting

**DOI:** 10.3390/ma13225074

**Published:** 2020-11-11

**Authors:** Serge Szmukler-Moncler, Cornelio Blus, David Morales Schwarz, Germano Orrù

**Affiliations:** 1 Oral Biotechnology Laboratory, Surgical Sciences Department, University of Cagliari, 09126 Cagliari, Italy; corblus@hotmail.com (C.B.); orru@unica.it (G.O.); 2 Private Practice, Calle Estadío, 1, 47006 Villadolid, Spain; dgms65@gmail.com

**Keywords:** titanium alloy, dental implant, surface characterization, etching, sandblasting, hydrogen concentration, concentration profile

## Abstract

Our purpose was to physically characterize the surface, and the subsurface, of a macro- and micro-textured titanium grade 5 dental implant surface obtained by etching only, without sandblasting. The topography, surface roughness, as well as the surface structure and subsurface distribution of elements, were determined by scanning electronic microscopy (SEM), non-contact profilometry, X-ray diffraction (XRD), and a concentration profile performed by Auger electron spectroscopy (AES). The hydrogen concentration in the implants was measured; the ability to generate nanostructures when stored in deionized water was also investigated. Under SEM, the surface resembled a sandblasted and etched titanium surface with its typical macro- and micro-texture; roughness was moderate with average roughness (Sa) 1.29 µm. No titanium hydride was found at the implant surface and no enrichment of any alloying element was identified at the surface and subsurface. Hydrogen concentration was 79 ppm, within the normative tolerance (<130 ppm). After storage in water for 6 months, densely packed finger-like nanostructures were observed. The clinical advantage of this textured titanium alloy surface is that it displays the typical macro- and micro-features of a moderately rough sandblasted and etched (SLA) titanium surface without leaving behind any foreign sandblasting material.

## 1. Introduction

Roughened surfaces of dental implants with an average roughness (Sa) in the 1–2 µm range are categorized as “moderately rough” surfaces [1]; they are considered to be optimized surfaces for bone ingrowth [1]. Acid etching is currently the most popular method used to texture the surface of dental implants [2,3,4]. With this chemical subtractive method, the only way to achieve a Sa roughness in the 1–2 µm range is to implement sandblasting (SB) prior to etching. Sandblasted-and-etched (SAE) surfaces always harbor remnant particles from the SB process [2,3,5]. Some manufacturers consider the residual alumina particles as a foreign material worth getting rid of; they are ready to forgo an optimized “moderately rough” surface and stick to a “minimally rough” micro-roughened surface with Sa <1 µm [2,6,7,8].

It has been recently shown that it was technically possible to achieve a “moderately rough” macro- and micro-textured surface on grade 5 titanium (TiAl6V4) through acid etching only, i.e., without any prior sandblasting [9]; strikingly the obtained topography was similar to the sandblasted one with large grit and acid attacked (SLA) surface obtained on cp titanium [2,10]. Saulacic et al. [11] stated that acid etching is typically not an appropriate treatment for α–β titanium (Ti) alloys. The reason is because its biphasic nature leads to an enrichment of vanadium from the β-Ti phase on the surface. Subsequently, careful characterization of the surface and subsurface of acid-etched titanium alloys is required to confirm or disprove this statement [12].

The aim of this paper was therefore to characterize the physical properties of this macro- and micro-textured biphasic α–β Ti alloy surface that has been obtained by etching alone, without sandblasting. The topographic characteristics of the surface, the presence of titanium hydride (TiH) and a concentration profile of the elements of the alloy were determined. The concentration of hydrogen (H) in the implants was measured to verify compliance with the H concentration requirements of DIN (17850, 17851) and ASTM (F67–89 and F468). These norms limit the concentration of hydrogen in finished products made out of titanium to 130 and 150 ppm, respectively. Finally, the ability of this etched surface to create nanostructures in a wet environment [13] was also checked.

## 2. Materials and Methods

Implants made of Ti grade 5 (Top DM, Ø 4.0 mm × 10 mm, Bioner, Sant Just Desvern, Spain) were acid etched according to a proprietary recipe without any prior sandblasting (BioEtch by Bioner). Their surface and subsurface was subsequently characterized.

### 2.1. Topographic and SEM Characterization

To characterize the features of the etched surface, observation was carried out with a ZEISS DSM-960A SEM at 20 kV (Zeiss, Jena, Germany); magnification varied between 20× and 2000×. Surface topography was further characterized by optical non-contact profilometry with a Bruker Contour GT-K1 (Bruker, Karlsruhe, Germany) on a 250 µm × 250 µm field with a Gaussian filter of 50 µm × 50 µm. Analysis was successively performed on 3 distinct implants in 3 consecutive valleys between the threads; average roughness (Sa), root mean square roughness of the surface (Sq), average distance between the highest peaks and lowest valleys of the surface (Sz), skewness of the height distribution (Ssk), kurtosis of the height distribution (Sku) and the developed surface (Sdr) were recorded.

### 2.2. X-Ray Diffraction

The presence of titanium hydride that appears on acid-etched cp Ti surfaces [12,14,15,16] or any other compound was investigated by XRD using a STOE θ/θ-Diffractometer (Stoe, Darmstadt, Germany) with a Cu Kα radiation (λ = 1.5418 Å, U = 40 kV, I = 40 mA) and a secondary beam monochromator of graphite (002). The 2θ scanned angle was 30–80°, step size of Δ2θ was 0.04° and counting time/step was 5 s. The investigated implant surface was set perpendicular to the radiation beam.

### 2.3. Concentration Profile of Elements

Enrichment by segregation of any element of the alloy toward the surface and into the oxide layer due to the specific proprietary etching process was investigated. A concentration profile of the elements of the alloy (Ti, Al, V), oxygen (O) and carbon (C) was obtained by Auger electron spectroscopy (AES) with a Perkin-Elmer, PHI 680 AES/SAM (Perkin-Elmer, Waltham, MA, US) while sputtering the surface with Ar+ ions; electron beam conditions were 10 keV and 10 nA. The argon ion gun used for depth profiling was operated at 2 keV and 1 μA at an angle of ca. 60° to surface normal; scan size was 1 mm × 1 mm, resulting in a sputtering rate of 12 nm/min to SiO_2_ target. Seventy abrasion cycles were implemented at 0.15 min/cycle.

### 2.4. Hydrogen Concentration

The concentration of H in the implants was measured according to ASTM E1147-09 [17] by the inert gas fusion thermal conductivity/infrared detection method; an RH 404 Leco (Leco Corp., St Joseph, MI, USA) was used. Five implants served to determine the concentration of hydrogen in the implants.

### 2.5. Ability to Form Nanostructures in Water

Immediately after etching and always kept wet, without allowing the surface to come into contact with air, 3 implants were stored in deionized water and left in situ for 6 months. The implants were then observed with a high-resolution SEM (ZEISS Ultra plus HR-SEM, Jena, Germany) at 2 kV and 25–150 k magnification to identify the presence of nanostructures that would have grown during their stay in deionized water.

## 3. Results

### 3.1. Topographic Characterization

**Figure 1:**
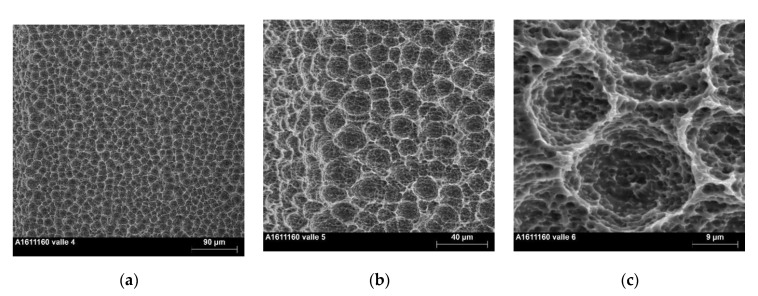
SEM micrographs of the implant surface. (**a**) Micrograph taken at 100× magnification. Note the homogeneous honeycomb macro-structure; (**b**) Micrograph taken at 500× magnification. Note the honeycomb-like macro-structure and the micro-pores for bone ingrowth, similar to a typical SLA surface topography; (**c**) Micrograph taken at 2000× magnification. Note the pores of the micro-texture superimposed on the rounded regular macro-structure.

**Figure 2:**
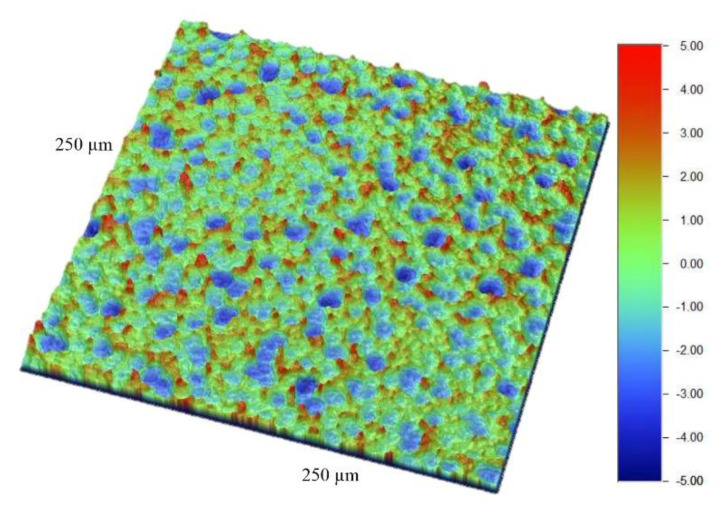
Surface roughness of the implant surface. Note the regular and homogeneous roughness structure with rounded valleys and peaks between the valleys.

SEM macrographs obtained at 100×, 500× and 2000× (Figure 1a–c) showed a regular honeycomb-like textured surface; the latter was already noted at low magnification (Figure 1a). At higher magnification, the macro-pore feature appeared regular and homogeneous, and the diameter range was 10–20 µm (Figure 1b,c). At 2000×, the micro-pores were well defined (Figure 1c) similar to the typical topography of SLA-treated cp titanium. No remnant of the β-Ti phase of the alloy that appears as white crystals [16,18] was found on the surface. Characteristics of the surface topography (Figure 2) were: Sa 1.29 ± 0.05 µm, Sq 1.61 ± 0.07 µm, Sz 11.23 ± 1.24 µm, Ssk 0.23 ± 0.05, Sku 2.79 ± 0.13 and Sdr 72.40 ± 7.26%.

### 3.2. X-Ray Diffraction

**Figure 3:**
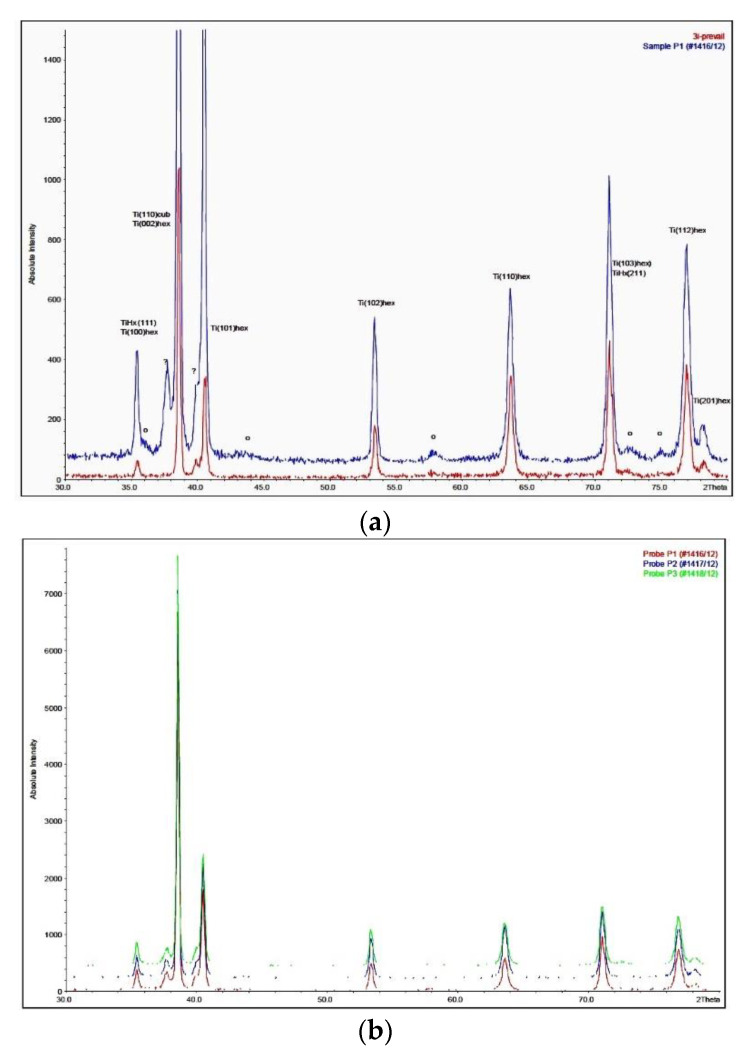
XRD diffraction of the implant surface. (**a**) XRD diffraction of the implant surface (blue line) compared to the diffraction of another etched Ti grade 5 alloy (Osseotite, red line). The peak at 38 degrees could not be identified, it was neither alumina nor hydride; (**b**) XRD diffraction on 3 distinct implants. The spectra were similar.

No titanium hydride peak was found on the XRD spectrum of the BioEtch-treated implants (Figure 3). It was similar to the XRD spectrum of other etched titanium alloy, devoid of titanium hydride (Figure 3a) [16]. The spectra were similar on all three analyzed implants (Figure 3b).

### 3.3. Concentration Profile of Elements

**Figure 4:**
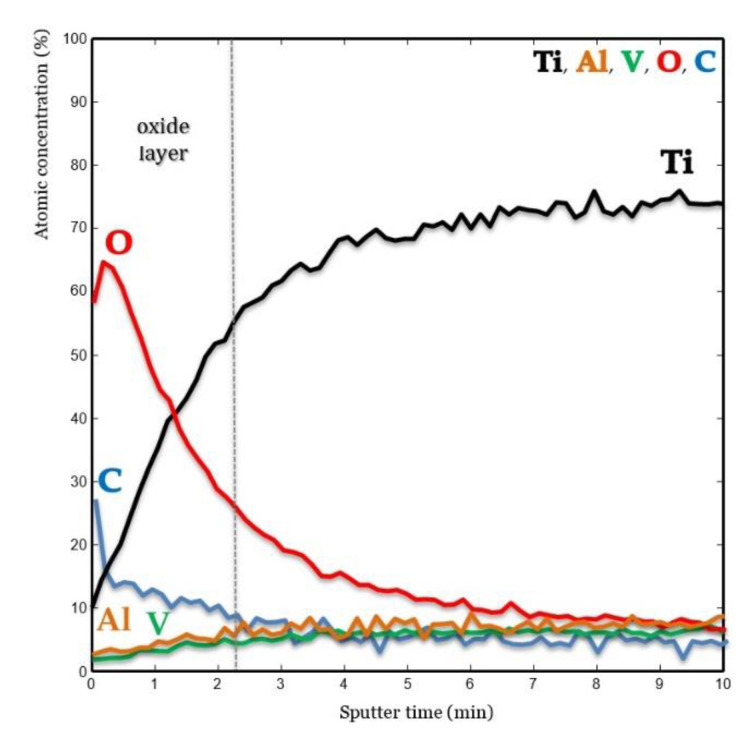
Concentration profile obtained by Auger electron spectroscopy (AES) and sputtering. Note the absence at the surface of a concentration of any of the alloying elements. On the contrary, a depletion of these elements was observed in the oxide layer of the implant surface.

The metallic compounds of the alloy, O and C, were followed over the 10 min long Ar^+^ sputtering (Figure 4). A steady stage of the concentration of the elements of the alloy was reached after 6 min of sputtering.

A high atomic concentration of C was measured at the outer surface of the oxide; the signal then steeply decreased toward the bulk of the alloy. A high concentration of O was also measured at the surface of the oxide; it then gradually decreased toward the bulk of the alloy. At the oxide outer surface, the O relative intensity was lower due to the presence of C. Concentration of Ti gradually increased from the surface until reaching the bulk of the alloy; its relative atomic concentration was lower within the oxide because of the presence of O. Concentrations of Al and V were lower in the oxide layer than in the bulk of the alloy. No increase in these elements within the oxide layer due to segregation was observed on any of the implant surfaces.

### 3.4. Hydrogen Concentration

The average concentration of H measured on five implants was 79 ± 1.0 ppm (min 75–max 81). It was within the tolerance of the ASTM and DIN norms, 150 and 130 ppm, respectively.

### 3.5. Ability to Form Nanostructures in Deionized Water

**Figure 5:**
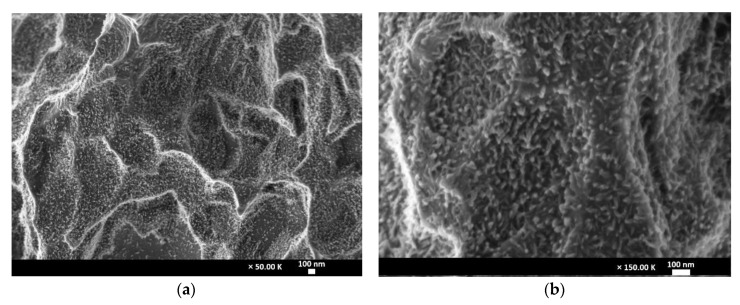
High resolution SEM micrographs of the implant surface. (**a**) Micrograph taken at 50k×. Note the dense package of the nanostructure all over the implant surface; (**b**) micrograph taken at 150k×. Note the finger-like nanostructures densely covering the surface.

After 6 months of storage in deionized water, dense finger-like nanostructures in the 10–30 nm range were found on the titanium alloy surface (Figure 5b); they were homogeneously covering the entire implant surface (Figure 5a).

## 4. Discussion

Surface treatments have been developed by manufacturers of dental implants to foster bone apposition at the implant surface and increase bone anchorage [19,20,21,22]. This has been demonstrated either by applying reverse torque [21,22], push-out [20] or tensile tests [23]. It has been suggested that a stronger anchorage in bone at an earlier time would allow for earlier and safer implant loading [21,22]. Several studies performed on sandblasted surfaces of distinct roughness showed that there is no advantage to struggle for a roughness beyond a Sa of 2 µm [24] or a Ra of 3.90 µm [23]. Actually, these studies showed that bone anchorage decreases when implant roughness exceeds these values. Wennerberg and Albrektsson [1,25] categorized dental implant surfaces into four distinct groups according to the level of roughness; they suggested that moderate roughness of a Sa in the 1–2 µm range is most desirable for a dental implant surface.

Several additive or subtractive methods can be implemented to reach a rough surface, but etching is the most popular one [3,4,25]. When etching alone is implemented, the average arithmetic roughness (Ra or Sa) remains always <1 µm, within the range of the “minimally rough” group [1]; this is the case of the Osseotite surface (Biomet-3i, Palm Beach Gardens, FL, USA) [6,7]. Depending on the measuring methods and devices, the Ra and Sa of this implant surface were 0.41 µm and 0.44 µm [26], 0.65 ± 0.04 µm [8], 0.68 µm [25], 0.72 ± 0.42 µm [27], 0.80 [28] and 0.96 ± 0.12 µm [2]. When the etching bath parameters used to obtain the SLA surface on Straumann implants [20] were applied on these very implants without, however, prior sandblasting, the Ra was 0.90 ± 0.11 µm [10], still within the “minimally rough” group with Ra < 1 µm [1,25].

Hot acid etching is a strong corrosion process that carves pores into the metallic implant surface; the result is a micro-roughness feature. Sandblasting prior to etching adds macro-roughness on top of the micro-roughness. Szmukler-Moncler et al. [10] compared, using the same implant design, the roughness obtained by sandblasting and etching versus etching alone. Etching alone leads to a Ra of 0.90 ± 0.12 µm, whereas the combination of macro- and micro-roughness obtained by SLA increased the Ra to 1.53 ± 0.11 µm [10], within the “moderately rough” surfaces group. After a 10-week healing period in the pig mandible, the anchorage of the SLA surface was stronger than the etched one by 49.3%, 157.3 ± 38.0 Ncm vs. 105 ± 25.1 Ncm. In addition, more bone was found attached to the SLA implant surface than to the etched one [10].

An inconvenience of the sandblasting process is that it always leads to remnant aluminum oxide particles on the surface [2,3,5,16]. In addition, the industrial process is not fully reproducible in terms of roughness and the number of alumina particles left behind [2,5]. Recently, Schüpbach et al. [5] compared nine distinct implant systems made of cp titanium, titanium grade 5 and a titanium–zirconium alloy, all were sandblasted and etched. According to implant system, the mean size of the particles varied from 159 to 1120 µm^2^; particles remaining on the titanium–zirconium (Ti–Zr) alloy implant showed an individual surface size covering up to 5900 µm^2^. Similarly, the number of particles varied according to the implant system from 0 to 905; again, the highest number of particles found on an implant surface was on the Ti–Zr alloy implant. Duddeck et al. [29] reported that alumina particles might cover up to 14.4% of the implant surface. Therefore, obtaining a macro- and micro-textured surface in the moderate roughness range by etching alone without sandblasting is highly desirable from a biological point of view. The present BioEtch surface with its macro- and micro-texture of Sa = 1.29 ± 0.05 µm achieves this requirement because the roughness is moderate and a total absence of alumina particles is warranted. To the best of our knowledge, this is the only surface on commercially available implants to offer this combination of a macro- and micro-texture generated without sandblasting; it can be considered as a unique technical know-how breakthrough.

**Figure 6:**
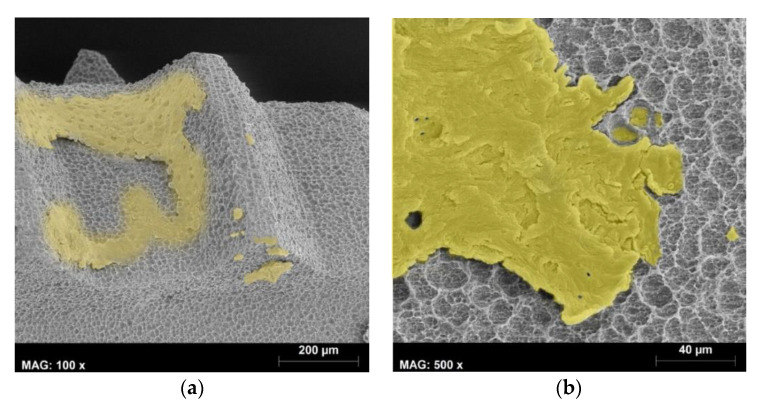
Bone attached to the implant surface after a mistaken reverse-torque applied to an implant after a 2-month healing period in the mandible. (**a**) SEM micrograph taken at 100×. Note the attached bone colored in yellow; (**b**) SEM micrograph taken at 500×. The attached bone is colored in yellow; note the bone remaining in the rounded macro-structure after the fracture of the implant–bone interface.

This micro- and macro-texture similar to the SLA feature should offer a good anchorage in bone. When the first implants with the BioEtch surface were placed into patients, osseointegration after 2 months of healing in the mandible and in the maxilla instead of 3 months in the mandible and 6 months in the maxilla was checked following the methodology suggested by Sullivan et al. [30] and Roccuzzo et al. [31]; resisting a reverse torque of 20 Ncm or 35 Ncm without inducing mobility would prove an appropriate implant osseointegration. A reverse torque of 50 Ncm was improperly applied during the verification of an implant. The mandibular implant rotated and became mobile; it was subsequently removed. SEM observation of this explant (with permission of the patient) showed bone attached to the surface (Figure 6a) and fractured bone portions remaining in the macro-pits where they had grown into (Figure 6b), similar to what was observed on Straumann SLA [10] or ITI TPS implants [22].

The XRD spectra did not display the presence of titanium hydride peaks that are usually obtained on cp Ti implants [10]. This is in line with a previous finding that only the surface of cp Ti implants are growing a TiH layer while Ti grade 5 implants did not [10]. The reason is that the α-Ti phase is the only phase in cp Ti implants and H solubility is limited in the α-Ti hexagonal close packed (hcp) structure. At a low concentration, H is accommodated at the octahedral interstitial sites and the balance is accommodated in the tetrahedral sites [32]. Above 20 ppm, however, H precipitates into TiH [33] and the α-Ti phase coexists with a non-stoichiometric δ-TiH_2-x_ phase [34]. On the other hand, the structure of the etched Ti grade 5 is biphasic, α + β; the β-Ti phase can amount up to 10%, depending on the heat treatment history [35,36]. H solubility in the β-Ti phase is much higher because the body-centered cubic (bcc) structure is able to accommodate more interstitial elements than the hcp structure. In Ti grade 5 and below 650 ppm, H is primarily concentrated in the β-Ti phase and very little goes to the α-Ti phase [35]. This explains why the 79 pmm of H measured in the etched implants did not lead to precipitation of TiH needles while this happens for cp Ti implants with similar H concentration [12,16].

H concentration in the BioEtched Ti grade 5 implants was 79 ± 1.0 ppm, within the requirements of the DIM (<130 ppm) and ASTM (<150 ppm) norms for finished products. It compares well with the H concentrations measured for the Ti grade 5 Osseotite and Biocom (MIS, Bar-Lev Industrial Park, Israel) implants, 55 and 106 ppm, respectively [16].

The concentration profile obtained by AES depth profiling showed a distribution of elements from the bulk to the oxide layer similar to the native oxide obtained on a Ti grade 5 surface polished with a 2400 grit SiC abrasive paper, cleaned in pure acetone and distilled water [37]. A contaminant layer of 27 at % C was present at the outermost layers of the surface. This relatively strong C signal is typical for adsorbed organic molecules on titanium surfaces that have been exposed to atmospheric conditions [3,8,27,38]. As expected, the O signal corresponding to the oxide layer decreased from the surface toward the bulk; it then remained constant at a lower level. In contrast, the Ti signal increased with sputtering time until a steady state was reached corresponding to the bulk material. A way to define the oxide thickness is to determine the sputtering depth at which the O signal has decreased 50% of its value in the oxide film [39]; this happened after 140 s of ion sputtering (Figure 4). The exact thickness of the oxide layer is difficult to determine since sputtering was performed on a roughened surface and not a plane reference one. The Al and V signals decreased from the bulk toward the oxide layer; they were similar to the native oxide layers grown on polished Ti grade 5 samples [37] or on an etched Ti grade 23 sandblasted and etched surface [12]. This means that no enrichment of any alloying element did occur at the surface or the subsurface as a result of this etching. Consequently, it is possible to affirm that this study disproves the statement of Saulacic et al. [11] that acid etching is typically not an appropriate treatment for α–β titanium alloys.

Noteworthy, when the etching conditions are strong enough, the α-Ti phase that is richer in Al and the β-Ti phase that is richer in V [12,17,40] dissolve simultaneously; they leave behind a homogeneous textured surface. This is the case for the BioEtch surface and for the etching performed on MIS implants [12,16]. Weaker etching conditions, such as the one applied to the Osseotite surface [7], are not able to simultaneously dissolve the α-Ti phase and get rid of the β-Ti phase that is richer in V. This explains why the Osseotite surface observed under SEM appears with sharp white grains looking like alumina particles deposited on the implant surface [16]. The latter are not remnants of alumina particles since no sandblasting was performed on that surface; they rather belong to the β-Ti phase that is richer in V and that is more acid resistant than the α-Ti phase [12,16,18,40].

Storage in standard deionized water without any other stringent conditions, such as nitrogen cover gas or saline storage [13,41], led to the formation of a homogeneous layer of densely packed finger-like nanostructures. It has been shown that the presence of this kind of nanostructure feature is able to stimulate earlier bone apposition when compared to the same treated surface left in air [13]. Noteworthy, the nanostructure density on the implant surface differs between cp Ti and the Ti–Zr alloy; it is dense for cp Ti and scattered for the Ti–Zr alloy [41]. On the present biphasic alloy, the nanostructure was found to extensively cover the implant surface, similar to the cp Ti surface. The effect of the size and density of the oxide nanostructures on osseointegration warrants further study.

## 5. Conclusions

This macro- and micro-textured Ti grade 5 surface obtained by etching only was found to be moderately rough, with a Sa in the 1–2 μm range. Its roughness was similar to SLA surfaces obtained on cp or Ti grade 5 implants without the need of prior sandblasting. Contrary to what has been previously suggested, acid etching is a suitable texturing process for the biphasic Ti grade 5 alloy; it does not lead to any vanadium or aluminum enrichment at the surface or the subsurface. No hydride layer was identified at the implant surface and the H concentration was within the normative requirements (<130 ppm). Finally, simple storage in deionized water was able to generate a homogeneous layer of densely packed finger-like nanostructures covering the entire implant surface.
